# The effectiveness of teaching reading comprehension strategies to higher education students in Chile

**DOI:** 10.3389/fpsyg.2026.1846429

**Published:** 2026-06-12

**Authors:** Miranda-Espinoza Jonathan Eduardo, Contreras-Saavedra Carla Elizabeth, María Jesús Sánchez-Cortés

**Affiliations:** 1 Facultad de Psicología y Humanidades, Universidad San Sebastián, Valdivia, Chile; 2 Laboratorio de Estudios Experimentales de la Comunicación, Facultad de Ciencias de la Rehabilitación y Calidad de Vida, Universidad San Sebastián, Concepción, Chile; 3 Facultad de Psicología y Humanidades, Universidad San Sebastián, Doctorado en Psicología y Salud Mental, Valdivia, Chile

**Keywords:** college student’s, educational intervention, reading comprehension, reading comprehension strategies, teaching effectiveness

## Abstract

**Introduction:**

Various studies show that many students enter higher education with insufficient performance in reading comprehension, which affects their disciplinary learning. Educational neuroscience suggests that guided, multimodal instruction in reading strategies can enhance the processes associated with reading comprehension. In this context, the importance of developing structured and culturally relevant interventions in higher education is emphasized.

**Method:**

This study assessed the effectiveness of teaching reading comprehension strategies to first-year college students enrolled in various degree programs at a university in southern Chile. This research employed a quantitative approach with a non-experimental, longitudinal, and descriptive design, using non-probabilistic convenience sampling, which included 404 students majoring in Psychology, Law, Obstetrics, Nursing, Medical Technology, and Nutrition and Dietetics. The LECTUM 7 Form A assessment tool was used to measure reading comprehension levels at two points in time: before and after a four-month intervention. During this intervention, short digital modules were provided to teach and practice reading comprehension strategies.

**Results:**

The results showed that, at the pre-test, only 51% of the students demonstrated average, high, or very high reading comprehension. After the intervention, this percentage increased to 75%—a 23% improvement compared to the pre-test (*p =* 0.001). Likewise, an increase was observed in the number of students who achieved normal, high, and very high reading comprehension performance, suggesting a positive effect of the explicit and systematic teaching of reading comprehension strategies in the context of higher education.

**Conclusion:**

These findings confirm the need to strengthen reading comprehension in the university context, with a focus on teaching strategies that enhance this skill and support students in their academic journey.

## 1 Introduction

Reading comprehension is a key competence in the academic and professional training of higher education students, since it allows them to access, process, interpret and critically evaluate information from various textual sources, especially in the framework of disciplinary studies. This skill is essential both for autonomous learning and for the development of analytical, critical and reflective thinking. However, various studies have shown that a significant number of students in Chile and Latin America enter higher education without having consolidated adequate levels of reading comprehension, which has an impact on their academic performance and the progression of their educational trajectories (Andrade and Utria, 2021; Amavizca Montaño and Álvarez-Flores, 2022; Cabrera-Pommiez et al., 2021; Curan, 2015; Errázuriz et al., 2020, 2022; Lillo-Fuentes et al., 2023; Makuc and Larrañaga, 2015; Miranda-Espinoza et al., 2025; Neira Martínez and Castro Yáñez, 2013; Neira et al., 2015; Núñez Cortés and Errázuriz Cruz, 2020; Sandoval et al., 2010; Velásquez et al., 2008; Vidal-Moscoso and Manríquez-López, 2016).

In this regard, several studies agree that a significant proportion of students enter higher education without having developed fundamental reading strategies during their school years, which directly affects their academic performance (Miranda-Espinoza et al., 2025; Neira et al., 2015; Errázuriz et al., 2020, 2022; Vidal-Moscoso and Manríquez-López, 2016). In light of this, several authors argue that institutions of higher education must take an active role in strengthening this skill, given that it is a necessary condition for equitable access to knowledge.

The contemporary approach to reading comprehension recognizes its multidimensional nature, incorporating dimensions such as explicit, implicit, textual, pragmatic, and critical comprehension (Cassany, 2006; Brizuela-Rodríguez et al., 2021; Cerda-Canales and Venegas, 2023; Gallego Ortega et al., 2019). This perspective has guided the design of diagnostic assessments and intervention programs aimed at identifying specific strengths and weaknesses in students, with the goal of proposing tailored reading strategies. Thus, while some students struggle to recognize communicative intentions or elements of textual cohesion, others require support in critical skills, such as comparing and contrasting viewpoints or identifying fallacies in arguments (Brizuela-Rodríguez et al., 2021; Cerda-Canales and Venegas, 2023; Ramírez, 2020; Miranda-Espinoza et al., 2025).

Taking these dimensions into account enables more precise and adaptive instruction, facilitating the transition from basic reading to complex and specialized academic reading. Furthermore, the literature indicates that the teaching of reading comprehension strategies in higher education should incorporate an active, reflective, and self-regulated approach, allowing students to construct meaning beyond the literal decoding of the text (Brizuela-Rodríguez et al., 2021; Franco et al., 2024; Reyna Zambrano and Castro Alcívar, 2023).

In coherence with this aspect, educational neuroscience has provided relevant theoretical frameworks for understanding certain cognitive processes associated with reading comprehension, particularly those related to attention, information integration, and self-regulation of learning. From this perspective, understanding a text involves coordinating processes of monitoring, inference, and the activation of prior knowledge, which has provided a foundation for pedagogical approaches focused on the planning, monitoring, and assessment of reading. Various studies have indicated that strategic and metacognitive instruction can enhance comprehension performance when organized in a structured and progressive manner (Cartwright et al., 2020; Follmer and Sperling, 2018). Likewise, previous research suggests that individual differences in reading comprehension are related to cognitive variables such as working memory and linguistic integration ability (Kim and Phillips, 2014). Consequently, this study draws on contributions from educational neuroscience as a complementary interpretive framework to inform pedagogical interventions aimed at progressively strengthening reading skills in higher education.

In this context, the effectiveness of metacognitive strategies as key mediators in the comprehension process has been highlighted. These strategies, which focus on monitoring, planning, and evaluating one’s own reading, enable students to take on a more active and conscious role when engaging with the text. Franco et al. (2024) observe that promoting self-reflection through guiding questions, goal-setting, and assessing their goal achievement significantly enhances comprehension levels. This assertion is supported by empirical evidence showing improvements in reading performance following the implementation of metacognition-based programs.

On the one hand, evidence shows that the use of structured reading strategies, such as academic reading workshops, is an effective tool for improving comprehension among college students. These approaches, grounded in active learning models, not only reinforce explicit and implicit reading comprehension skills but also promote self-regulation, critical reflection, and the transfer of learning to other contexts. Studies such as those conducted by Asensio Pastor (2019) and Guerra-García et al. (2022) have demonstrated that workshops centered on the analysis of academic texts, combined with opportunities for self-evaluation and group discussion, have a positive impact on students’ appropriation of academic styles and the strengthening of their reading skills.

On the other hand, evidence suggests that reading comprehension at the university level cannot be limited to a literal understanding of the content, but must meet the growing cognitive demands of analysis, synthesis, argumentation, and the transfer of knowledge (Amavizca Montaño and Álvarez-Flores, 2022; Brizuela-Rodríguez et al., 2021; Cevallos Bone, 2023; Neira et al., 2015). Students who have not properly mastered strategies for inference, textual integration, or critical assessment tend to struggle with acquiring disciplinary knowledge, which directly affects their academic performance and their participation in independent learning activities. For this reason, several authors propose that reading comprehension strategies should be systematically integrated into the university curriculum, especially during the early years of study, a stage in which the foundations of academic thinking are established.

Following this line of thinking, interventions that combine multiple reading strategies with the use of digital technologies and visual resources-such as graphic organizers, interactive modules, gamification, or virtual guided-reading platforms-have been found to be more effective (Domínguez et al., 2024; Franco et al., 2024; Sánchez et al., 2023; Reyna Zambrano and Castro Alcívar, 2023). These proposals have proven to be particularly effective in enhancing global, inferential, and critical comprehension by facilitating the visual representation of information, promoting hierarchical relationships between ideas, and increasing motivation toward academic texts. In this way, the use of technology serves as a means to enrich reading practices, adapt them to today’s learning environments, and address the cognitive characteristics of college students.

From this perspective, reading strategies must be tailored to the types of texts used in each discipline, considering their linguistic, argumentative, and epistemological features. This approach has resulted in the introduction of curricular interventions in first-year courses. These interventions integrate comprehensive, critical, and metacognitive reading activities as part of the learning objectives, which has been demonstrated a favorable impact on students’ academic performance (Domínguez et al., 2024; Cassany, 2006; Carlino, 2005).

Given the background information presented, the aim of this study is to determine the effectiveness of teaching reading comprehension strategies to first-year college students enrolled in various degree programs at a university in southern Chile. Also, this study aims not only to identify the impact of the intervention on reading comprehension performance, but also to contribute to the pedagogical discussion regarding the need to systematically integrate the teaching and development of reading comprehension into higher education practices.

## 2 Method

The study employed a quantitative approach using a single-group quasi-experimental design with pre-test and post-test assessments, conducted longitudinally to describe the level of reading comprehension in students’ initial and final performance following instruction in reading comprehension strategies. This study utilized a non-probabilistic convenience sample to facilitate data collection within a university setting, selecting participants based on their availability. Due to the absence of a control group, the results must be interpreted with consideration of the limitations inherent in this type of design, particularly regarding the exclusive attribution of the observed changes to the implemented intervention.

### 2.1 Participants

The sample consisted of 404 university students, of whom 74% were women and 26% were men. In terms of educational background, 56% attended subsidized private schools and 44% attended municipal schools. Regarding their fields of study, 22% were in Nursing, 15% in Obstetrics, 10% in Nutrition and Dietetics, 11% in Medical Technology, 19% in Law, and 23% in Psychology.

### 2.2 Instruments

The instrument used was the LECTUM 7, Form A and B, a validated psychometric battery for high school students (Riffo et al., 2011) that has previously been used in university settings (Neira et al., 2015; Miranda-Espinoza et al., 2025) and has proven to be a useful tool for assessing reading comprehension levels in this age group. Given that the participants in this study are first-year students in various degree programs and the lack of standardized instruments for this population in Chile, we chose to use the LECTUM 7 as a valid tool to obtain an approximation of reading comprehension performance levels at this educational level.

This test evaluates five dimensions of reading comprehension: explicit, implicit, textual, pragmatic, and critical. Each item is scored on a performance scale that takes into account the type of school the student attends, whether it is a subsidized private school or a public school.

The LECTUM 7 provides five categories of performance scales, which allow for the evaluation of an individual’s or a group’s performance relative to a reference population; in other words, the interpretation of the score will vary depending on the school where the participant is currently enrolled or previously attended (see Table 1).

**Table 1:** Reading comprehension performance categories according to LECTUM 7.

Performance categories	Raw score range for government institutions	Raw score range for partially government-subsidized and private institutions
Very low	0	Menor 4
Low	1–7	4–11
Normal	8–21	12–28
High	22–28	29–28
Very high	Greater than 28	Greater than 36

LECTUM-7 Form A and B were originally developed and validated for secondary school students. Their use in the present study was justified by the absence of equivalent standardized instruments for the Chilean university population and by their previous use in the assessment of reading comprehension in Spanish.

Because the platform used provides aggregated scores (total score and dimension scores) rather than item-level responses, it was not possible to recalculate classical indicators of internal consistency or to conduct a complete reevaluation of the instrument’s factorial structure in this sample. Nevertheless, the distribution of the obtained scores was examined to explore potential ceiling effects, without observing a concentration of scores near the maximum value of the instrument.

The data were analyzed using descriptive statistics with the statistical software JAMOVI version 2.6.5.

### 2.3 Reading comprehension strategies

After administering the LECTUM, short video clips were sent via email over 13 weeks (one capsule per week), incorporating various strategies for improving reading comprehension. Students could watch the clips and complete the suggested activities on their own, adapting them to their own schedules and needs.

In total, 13 video clips were sent, each lasting between 3 and 6 min over 13 weeks (see Table 2).

**Table 2:** Reading comprehension strategies by dimension.

Dimension of reading comprehension	Reading comprehension strategies	Definition
Explicit textual	Approaching the text	It is the reader’s ability to identify and understand how a text is organized in terms of its different parts or sections; this includes recognizing the introduction, body, and conclusion, as well as identifying cues such as headings, subheadings, paragraphs, and connectors (Neira et al., 2015).
Implicit	Word deduction	It involves inferring the meaning of words in a text based on its general and specific contexts, where the general context refers to the overall idea of the text and the specific context refers to the ideas that come together to form the text’s structure and content (Neira et al., 2015).
Pragmatic critical	Questioning the text	This involves distinguishing between what the author says and what the reader thinks (Neira et al., 2015).
Explicit	Underlined	A highlighting technique that identifies key pieces of information to facilitate selection and review; its effectiveness is enhanced when it is combined with summarization and note-taking, rather than being limited to mechanical highlighting (Salcedo Murillo, 2023).
Explicit	Summary	A concise yet faithful paraphrase of the content that preserves the main ideas and the text’s overall structure; it aids in understanding and assimilating the information (Salcedo Murillo, 2023).
Implicit pragmatic critical	Synthesis (Overview)	It involves the ability to combine main ideas and relevant details from a text. It allows one to select key information, preserving the original logic while presenting it in a more accessible and understandable way (Del Pino-Yépez et al., 2019).
Explicit textual	Hierarchization of information	Organizing ideas from broader concepts to specific details to clarify the text’s macrostructure and facilitate its retention and transfer (Montes-Salas et al., 2014). This module examined visual representation strategies that externalize the structure of the content and the relationships between concepts, thereby facilitating deep understanding and recall.
Implicit textual	Concept map	A propositional and hierarchical representation of concepts linked by linking words; it makes semantic relationships explicit and promotes meaningful learning (Montes-Salas et al., 2014).
Textual	Summary table	A bracket outline that summarizes and organizes topics, subtopics, and details to visualize the text’s hierarchical estructure (whole-part relationship) (Montes-Salas et al., 2014).
Implicit critical	Minds maps	A radial diagram centered on a core idea, from which branches with associated words or images extend; useful for activating knowledge, generating ideas, and organizing content in a flexible manner (Peña Rañileo et al., 2021).
Implicit pragmatic	Flowchart	A sequential representation of the steps and decisions in a process, which helps to understand procedures and causal relationships within the content (Montes-Salas et al., 2014).
Textual pragmatic critical	Comparative table	A matrix that compares categories and attributes to identify similarities and differences between concepts or cases, facilitating synthesis and decision-making (Montes-Salas et al., 2014).

The clips were organized according to the criteria established at the outset. To begin and provide context for the students, an introductory clip was sent that provided general information about reading comprehension strategies.

This classification of the modules was developed by integrating the multidimensional approach to reading comprehension (Cassany, 2006; Brizuela-Rodríguez et al., 2021; Cerda-Canales and Venegas, 2023), along with the psycholinguistic and metacognitive foundations used in the study, given that the selected strategies were not exclusively oriented toward the development of explicit and textual skills, but rather sought to progressively address implicit, pragmatic, and critical dimensions of reading comprehension, taking into account the multidimensional nature of the construct.

To create the capsules, the following dimensions of reading comprehension were taken into account.

To make the videos easier to view and encourage their visualization, a consistent structure was maintained across all of them (see Table 3).

**Table 3:** Structure of video clips featuring reading comprehension strategies.

Structure	Contents	Definition
Presentation	Name of strategies	The presentation of the number and the strategy to be implemented during the informative capsule took place.
Introduction	Objectives	At this point, the objectives to be achieved in each of the informative capsules were specified.
Main body	Definition methodology activity Example activity	The strategy was defined in theory, and a methodology for its implementation was provided. An activity was conducted using a sample text. The student received an example of the strategy applied to a text. The student received another text to practice applying the strategy. In addition, questions were provided to guide the workbook.
Appendix	Optional readings.	The informative capsules were accompanied by one or two supplementary articles for students to review and apply the strategies, supplementing the information provided.

### 2.4 Reading comprehension strategies protocol

Each student received a weekly reading comprehension module for 13 consecutive weeks, delivered via the university’s institutional email system. The videos ranged in length from a minimum of 3 min to a maximum of 6 min. The modules were organized progressively, moving from strategies focused on textual recognition and organization toward inferential, pragmatic, and critical strategies, consistent with the multidimensional nature of reading comprehension.

Each segment featured a presenter responsible for introducing the strategy, the learning objectives, and the implementation of the reading comprehension strategy. The presenter served as a strategic model, explaining step-by-step how to apply each strategy through guided examples and the guided completion of activities. During the session, students observed the definition of the strategy, its implementation methodology, and a text-based activity used as a model or scaffold to illustrate its use. Subsequently, a second example of the strategy’s application was presented using a different text.

Later on, students were invited to apply the reading comprehension strategy to a new text included in the same module, with the aim of independently practicing the skill they had learned. To this end, the module included guiding questions, strategic modeling, guided examples, and step-by-step solutions to the activities. Upon completing this exercise, the student viewed the expected solution to the activity. Additionally, each participant had access to two other supplementary texts as additional material for independent practice. Consequently, each student completed at least one guided practical application and one independent application of each reading comprehension strategy taught.

The videos were viewed asynchronously and independently at home, according to each student’s own schedule. However, faculty members from the various programs conducted a general monitoring of access to and viewing of the video segments based on self-reports submitted by the students themselves. Due to the asynchronous nature of the intervention, it was not possible to individually verify the actual viewing time or the level of independent completion of all the proposed activities.

It should be noted that the activities proposed in the modules were not graded, and participation was voluntary. Furthermore, due to the asynchronous nature of the program, it was not possible to accurately determine the individual completion rate for all modules.

### 2.5 Research procedure

This research was approved by the Scientific Ethics Committee of the University of San Sebastián in Valdivia (ID 09–24).

Regarding the procedure, first, the participant received information about the study and signed an informed consent form to authorize their participation in the study. Second, data were collected in person by administering the LECTUM 7 questionnaire form A to each student; this process took place in the university computer labs.

Third, guided instruction in reading comprehension strategies was implemented; these were sent to students weekly via email over 13 weeks, consisting of a total of 13 short video clips. Finally, a second assessment was conducted in person, again using the LECTUM 7 form B, to measure the impact of the reading comprehension strategy instruction.

### 2.6 Data analysis design

First, descriptive statistics were calculated for the initial assessment, taking into account the total raw score of the test and the interpretation based on LECTUM 7, which includes scores for the explicit, implicit, textual, pragmatic, and critical dimensions of reading comprehension, and classifying each student into different categories (see Table 1).

Second, the Shapiro–Wilk test was used to determine whether the data followed a normal distribution. Since the data did not follow a normal distribution, the nonparametric Wilcoxon test was then used to measure the impact of the intervention before and after the teaching of reading comprehension strategies. During the process, the statistical software SPSS and JAMOVI were used to process, describe, analyze, and compare the data obtained.

## 3 Results

### 3.1 Reading comprehension performance on the initial assessment

**Figure 1:**
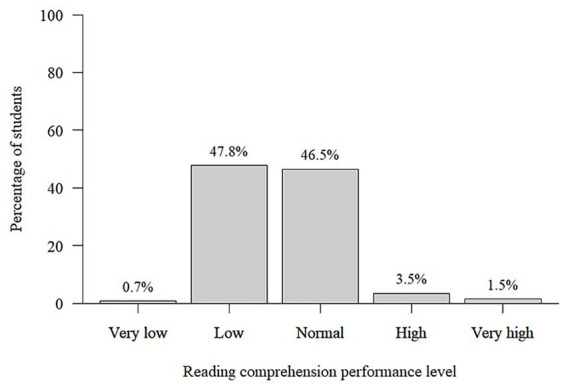
Reading comprehension performance on the initial assessment. Created by the author.

Figure 1 illustrates the distribution of students according to their reading comprehension performance, classified into five categories: very low, low, normal, high, and very high, in accordance with the criteria of the LECTUM 7 assessment tool. The graph shows that 48% of students have low reading comprehension performance, and 1% have very low performance, representing 49% of students with performance below expectations. On the other hand, 47% fall into the average category (normal), 3% into the high category, and 1% into the very high category, meaning that 51% of students perform at an adequate level.

Concerning Table 3 and the dimensions assessed, better performance was observed in explicit and textual reading comprehension, indicating that most students have greater facility in identifying literal information and understanding the internal structure of texts. However, the implicit, pragmatic, and critical dimensions showed the lowest scores, suggesting difficulties in inferential reasoning, contextualization, and critical analysis of content (Table 4).

**Table 4:** Descriptive statistics on the dimensions of reading comprehension in the initial assessment of first-year college students.

Dimensions of reading comprehension	Mean-(SD)	SE	Median	Mode	Variance	Min-Max
Direct score	15.84 – (6.8)	0.33	16	11	46.64	0–38
Explicit	8.65 – (4.0)	0.20	8	6	16.52	0–22
Implicit	7.23 – (3.4)	0.16	7	5	11.54	0–17
Textual	12.71 – (5.7)	0.28	13	8	33.21	0–33
Pragmatic	1.68 – (1.1)	0.05	2	1	1.30	0–5
Critical	1.46 – (1.0)	0.05	1	1	1.11	0–4

SD, Standard deviation; SE, Standard Error; Min, Minimum; Max, Maximum.

### 3.2 Reading comprehension performance on the final exam

**Figure 2:**
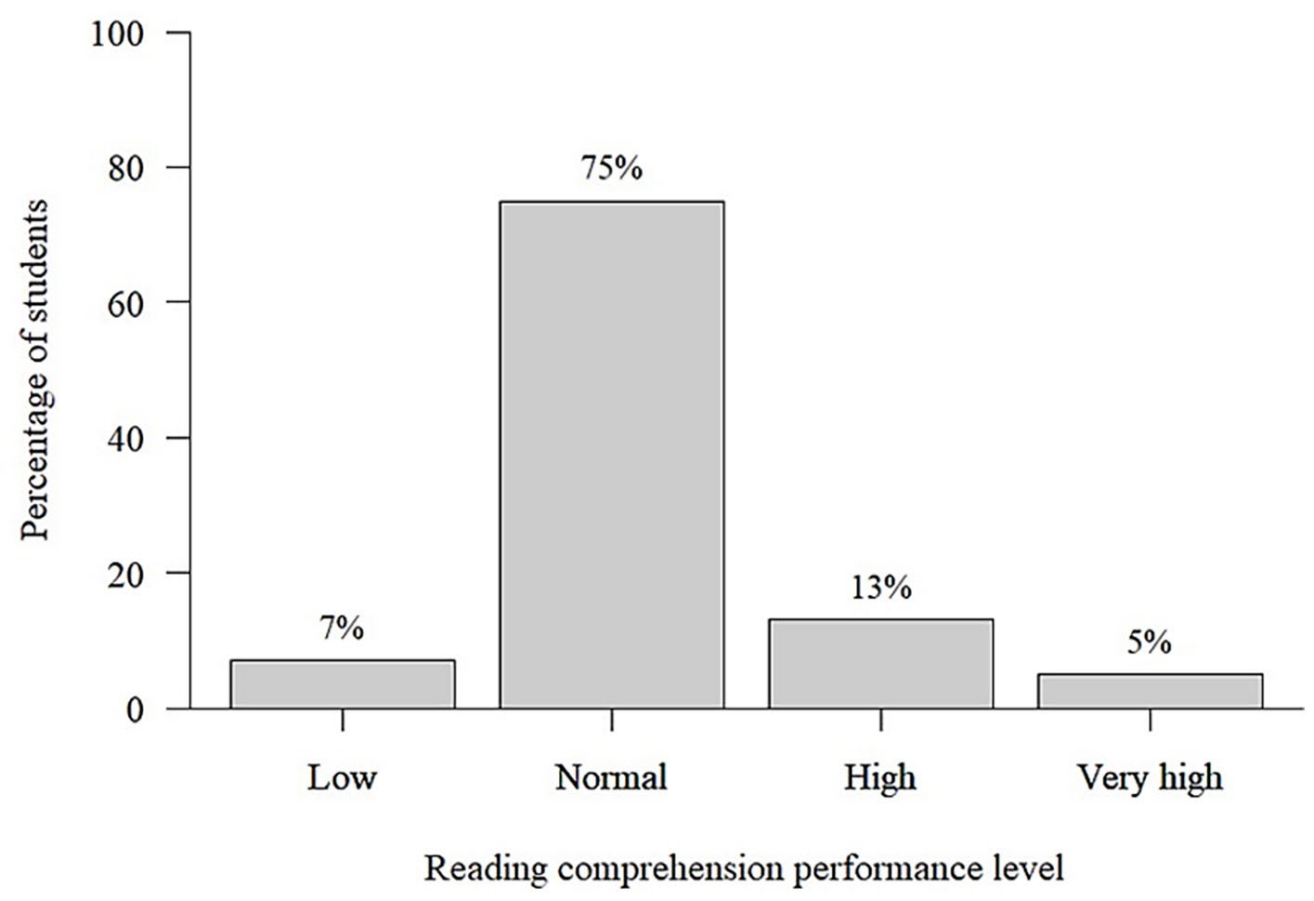
Reading comprehension performance on the final exam. Created by the author.

Figure 1 illustrates the distribution of students according to their reading comprehension performance, classified into five categories: very low, low, normal, and high, in accordance with the criteria of the LECTUM 7 assessment tool. The graph shows that 0% of students scored in the very low reading comprehension category, while 25% of students are in the low category, with performance below expectations. On the other hand, 57% are in the normal category, 13% in the high category, and 5% in the very high category; that is, 75% of the students demonstrate adequate reading comprehension performance, representing a 23% increase compared to the initial assessment (Figure 2).

### 3.3 The effectiveness of teaching reading comprehension strategies using the Wilcoxon test

To evaluate the effectiveness of teaching reading comprehension strategies, the Wilcoxon signed-rank test was applied, given that the data did not follow a normal distribution, as verified by the Shapiro–Wilk test (*p* < 0.001 across all dimensions). This test allowed for a comparison of students’ performance before and after the instruction in reading comprehension strategies, across the various dimensions of reading comprehension: direct score, explicit, implicit, textual, pragmatic, and critical comprehension (Table 5).

**Table 5:** Descriptive statistics on the dimensions of reading comprehension in the final assessment of first-year college students.

Dimensions of reading comprehension	Mean-(SD)	SE	Median	Mode	Variance	Min-Max
Direct score	17.90 – (6.3)	0.32	17	11	40.11	6–38
Explicit	10.35 – (5.4)	0.17	10	9	12.36	4–22
Implicit	7.56 – (3.1)	0.15	7	5	9.56	1–17
Textual	14.79 – (5.3)	0.27	14	13	28.87	4–33
Pragmatic	1.69– (1.2)	0.06	2	1	1.31	0–5
Critical	1.48– (1.1)	0.05	1	1	1.12	0–4

SD, Standard deviation; SE, Standard Error; Min, Minimum; Max, Maximum.

The results showed (see Table 6) statistically significant differences in the total score and in the explicit, implicit, and textual dimensions (*p <* 0.001), indicating that the intervention had an effect on these levels of reading comprehension. Overall, the total score showed a significant increase following the implementation of the reading comprehension strategies (*p <* 0.001).

**Table 6:** Comparison of pre- and post-instruction results for reading comprehension strategies across their various dimensions using the Wilcoxon test.

Dimensions of reading comprehension	Median (max-min) Initial assessment	Median (max-min)Final assessment	W	*p* value	r	Magnitude
Direct score	16 (11–20)	17 (14–22)	15051.0	<0.001*	0.6466	Large
Explicit	8 (5–11)	10 (8–12)	33331.5	<0.001*	0.7539	Large
Implicit	7 (5–9)	7 (5–9)	18307.5	<0.001*	0.1756	Small
Textual	13 (8–17)	14 (11–18)	16718.0	<0.001*	0.65082	Large
Pragmatic	2 (1–2)	2 (1–2)	0.0	0.174	−0.08617	Very small
Critical	1 (1–2)	1 (1–2)	2.00	0.345	−0.04981	Very small

*p < 0.05. Effect sizes (r) were interpreted according to Cohen’s (1988) criteria: very small < 0.10, small ≥ 0.10, medium ≥ 0.30, large ≥ 0.50. 95% CI are reported as post-pre differences. In pragmatics and criticism, CI is not reported due to the presence of ties and zeros in the differences. p-values were calculated with continuity correction due to the presence of ties.

Regarding effect size, estimated using r, a large effect was observed on the total score (*r =* 0.647), as well as on the explicit (*r =* 0.754) and textual (*r =* 0.651) dimensions; a small effect on the implicit dimension (*r* = 0.176); and very small and non-significant effects on the pragmatic (*r =* 0.086; *p =* 0.174) and critical (*r =* 0.050; *p =* 0.345) dimensions.

These findings suggest that the intervention using reading comprehension strategies had an impact on students’ reading comprehension levels, proving effective in improving explicit access to written information and promoting the development of complex and integrative reading skills, which are essential for the academic performance of first-year students in health-related degree programs. However, it should be noted that while there are increases in the mean scores for pragmatic and critical reading comprehension, these do not represent statistically significant differences or a relevant effect size, which can be explained by the cognitive complexity these dimensions require and the amount of exposure time needed for their development.

## 4 Discussion

### 4.1 Reading comprehension in first-year university students

The objective of this research was to determine the effectiveness of the teaching of reading comprehension strategies in higher education students who enter the first year in different university careers in southern Chile, through an intervention based on digital modules that provide guided instruction in reading comprehension strategies. The results showed statistically significant differences in most of the dimensions evaluated (*p <* 0.001), except for pragmatic and critical understanding, which confirms the positive impact of the intervention implemented. These findings are aligned with the literature that describes gaps in higher education entry and the need to systematically strengthen reading comprehension at this formative stage (Amavizca Montaño and Álvarez-Flores, 2022; Errázuriz et al., 2020, 2022; Miranda-Espinoza et al., 2025).

### 4.2 Reading comprehension from a multidimensional perspective

From a psycholinguistic perspective, the results reinforce the idea that reading comprehension constitutes a processual, dynamic, and trainable competence, which does not develop spontaneously, but requires modeling, scaffolding, and guided practice (Makuc and Larrañaga, 2015). The observed pattern, better performance in explicit and textual dimensions, together with greater challenges in implicit, pragmatic, and critical dimensions, is consistent with the multidimensional nature of the reading comprehension construct (Brizuela-Rodríguez et al., 2021; Cerda-Canales and Venegas, 2023).

Indeed, while literal identification and textual organization skills can be strengthened through structured strategies such as underlining, hierarchizing, and the use of graphic organizers, the pragmatic and critical dimensions require processes of greater cognitive depth, such as the construction of complex inferences, the activation of prior knowledge, and argumentative evaluation.

This finding can be interpreted in the light of Kintsch’s (1998) construction-integration model, which states that deep understanding depends on the elaboration of a situation model, which implies integrating textual information with previous schemas and metacognitive mechanisms. Along these lines, although the intervention achieved significant advances in the processing of the base text (explicit, textual and even partially implicit), the pragmatic and critical dimensions that are associated with the assessment of communicative intention and argumentative judgment require more consolidation time and more dialogic and contextualized reading experiences (Carlino, 2005; Cassany, 2006).

### 4.3 Metacognition and Reading comprehension strategies

Likewise, the results dialog with the evidence that underlines the role of metacognition as a mediator of reading performance. As Franco et al. (2024) and Reyna Zambrano and Castro Alcívar (2023) point out, goal planning, comprehension monitoring, and achievement evaluation allow the student to take an active role in the text, favoring sustained improvements in performance. The incorporation of brief, structured, and progressive capsules, along with autonomous activities and visual resources, is also aligned with proposals that highlight the effectiveness of technologically mediated interventions to strengthen reading skills in higher education (Domínguez et al., 2024; Sánchez et al., 2023).

### 4.4 Reading comprehension and academic literacy

From the perspective of academic literacy, the results support what Núñez and Errázuriz et al. (2020) propose, who emphasize that the university must assume responsibility for the development of discursive competencies manifested in reading comprehension specific to each discipline. Similarly, Lillo-Fuentes et al. (2023) warn that mastery of complex discursive and reading comprehension practices is a requirement for the appropriation of specialized knowledge, while Sandoval et al. (2010) and Velásquez et al. (2008) show that reading difficulties in university admission affect various areas of knowledge transversally.

### 4.5 Challenges in pragmatic and critical dimensions

As for the absence of significant differences in the pragmatic and critical dimensions, this can be explained both by the cognitive complexity involved and by the need to integrate these skills more systematically into authentic disciplinary tasks, where the analysis of arguments, the detection of fallacies and the comparison of perspectives are recurrent practices (Leyva Ato et al., 2022).

Therefore, the absence of significant changes in pragmatic and critical comprehension may indicate that these dimensions require more prolonged, contextualized, and dialogic reading experiences, as they involve processes of argumentative evaluation, contextual interpretation, and disciplinary integration that are cognitively more complex. Likewise, although the intervention incorporated strategies related to these dimensions, there was a greater concentration of activities focused on explicit recognition and textual organization, which could explain the greater effects observed in these areas. Consequently, future interventions could incorporate a greater concentration of contextualized argumentative exercises within texts specific to the different disciplinary areas, thereby facilitating transfer to real academic settings.

### 4.6 Theoretical contributions from educational neuroscience

On the other hand, Educational neuroscience has suggested that reading comprehension processes may involve varying levels of cognitive complexity, noting that skills related to information identification and textual organization tend to develop more rapidly than those associated with critical interpretation and argumentative evaluation (Cartwright et al., 2020; Follmer and Sperling, 2018; Kim and Phillips, 2014). From this theoretical perspective, the results of the present study may suggest the need for longer-term, gradual interventions that promote the progressive strengthening of inferential and evaluative skills.

### 4.7 Pedagogical implications for higher education

In pedagogical terms, the results confirm that the explicit and systematic teaching of reading strategies during the first year of university is a relevant measure to promote equity in access to knowledge and support the transition to academic culture (Errázuriz et al., 2020; Carlino, 2005). The intervention developed not only had a remedial character, but also promoted higher-order skills, consolidating the foundations of academic thinking in students who face high cognitive and disciplinary demands, particularly in careers in the health area (Riffo and Contreras, 2012; Neira et al., 2015).

In summary, this intervention demonstrates that it is possible to design pedagogical proposals with a solid theoretical basis and empirical support, which articulate principles of psycholinguistics, textual linguistics and pedagogy to respond to the current challenges of higher education. However, it is necessary to deepen interventions of longer duration and with longitudinal follow-up, which allow evaluating the sustained impact on critical understanding and academic performance in the medium and long term.

## 5 Conclusion

The results of this research allow us to conclude that the guided teaching, systematic and technologically mediated teaching of reading comprehension strategies produces significant improvements in the reading performance of first-year students of higher education. The observed increase in the overall score and in the explicit, implicit and textual dimensions confirms that reading comprehension is a trainable competence that can be strengthened through structured interventions, which supports the approaches of psycholinguistics and processual models of comprehension.

In coherence with the multidimensional approach to reading, the findings show that the dimensions associated with literal recognition and textual organization demonstrate greater plasticity in medium-term interventions, whereas pragmatic and critical dimensions, linked to the construction of deep situational models, argumentative evaluation, and disciplinary integration, require more systematic, prolonged, and contextualized instructional experiences within authentic academic tasks. This finding is consistent with the construction-integration model and the contributions of academic literacy, which suggest that university reading involves the progressive appropriation of specialized discursive practices.

Likewise, the incorporation of short and structured digital capsules proved to be a viable and replicable modality, aligned with contemporary proposals that integrate technology, metacognition and visual resources to favor reading self-regulation. From the perspective of educational neuroscience, these results can be interpreted as consistent with theoretical proposals that emphasize the importance of strategic, gradual, and multimodal instruction in promoting processes associated with reading comprehension and self-regulation of learning in higher education.

Overall, this study provides relevant empirical evidence to support the systematic incorporation of the teaching of reading comprehension in the university curriculum, especially during the first year, as a key strategy to promote equity and academic success in higher education.

### 5.1 Limitations

Among the study’s limitations are the absence of a control group, the use of non-probabilistic convenience sampling, and the asynchronous and self-directed nature of the intervention, which made it difficult to individually verify students’ level of participation. Furthermore, although the LECTUM 7 has been previously used with a Chilean university population, it was originally designed as an instrument for secondary education; therefore, further development of psychometric evidence specific to higher education is required.

It is proposed as a future line to deepen longitudinal interventions that incorporate disciplinary argumentative tasks and academic monitoring, as well as to explore the differential impact according to career, reading profile and sociodemographic variables.

## Data Availability

The raw data supporting the conclusions of this article will be made available by the authors, without undue reservation.
