# Correction: The effectiveness of teaching reading comprehension strategies to higher education students in Chile

**DOI:** 10.3389/fpsyg.2026.1934437

**Published:** 2026-07-21

**Authors:** Miranda-Espinoza Jonathan Eduardo, Contreras-Saavedra Carla Elizabeth, María Jesús Sánchez-Cortés

**Affiliations:** 1 Facultad de Psicología y Humanidades, Universidad San Sebastián, Valdivia, Chile; 2 Laboratorio de Estudios Experimentales de la Comunicación, Facultad de Ciencias de la Rehabilitación y Calidad de Vida, Universidad San Sebastián, Concepción, Chile; 3 Facultad de Psicología y Humanidades, Universidad San Sebastián, Doctorado en Psicología y Salud Mental, Valdivia, Chile

**Keywords:** college student's, educational intervention, reading comprehension, reading comprehension strategies, teaching effectiveness

Funder name incorrect

An incorrect Funding statement was provided. The correct funding statement reads:

## Funding

The author(s) declared that financial support was received for this work and/or its publication. This study was supported by Vicerrectoría Académica and Centro de Investigación para la Educación Superior (CIES), Universidad San Sebastián, through Fondo USS-FIN-2026_APC_VRA-005.

The original version of this article has been updated.

